# Latent profiles of spiritual care competence among Chinese nursing undergraduates: Correlations with spiritual care cognition and meaning of life

**DOI:** 10.1371/journal.pone.0342051

**Published:** 2026-02-06

**Authors:** Yang Guo, Ruonan Wang, Anqi Li, Jie Yao, Lingkun Wang

**Affiliations:** 1 Department of Nursing, School of Medicine, Shaanxi Institute of International Trade & Commerce, Xianyang, Shaanxi Province, China; 2 Department of Nursing, Medical College, Xijing University, Xi’an, Shaanxi Province, China; 3 Department of Nursing, Medical College, Xi’an Peihua University, Xi’an, Shaanxi Province, China; 4 Nursing College, Shaanxi University of Chinese Medicine, Xi’an, Shaanxi Province, China; 5 Department of Emergency, Shandong Provincial Hospital Affiliated to Shandong First Medical University, Jinan, Shandong Province, China; Universidad Complutense de Madrid, SPAIN

## Abstract

**Background:**

Spiritual care occupies a special position in overall care and improving the quality of services provided to patients. However, China’s higher nursing education is still in its infancy. The purpose of this research is to explore the heterogeneity of spiritual care competence of undergraduate nursing students in China.

**Methods:**

A multicenter cross-sectional design was employed. From July 2024 to February 2025, a convenience sample of 1,224 undergraduate nursing students was recruited from four nursing colleges in the Guanzhong region of Shaanxi Province, China. Data were collected using the Chinese versions of the Spiritual Care Competence Scale, the Spiritual Care Cognition Scale, and the Meaning in Life Questionnaire. Descriptive and correlational analyses were performed using SPSS version 27.0, while latent profile analysis and ordinal logistic regression were conducted using Mplus version 8.0.

**Results:**

The mean total score for spiritual care competence was 77.61 ± 14.61. Latent profile analysis identified three distinct profiles: a low-competence group (47.1%, 62.3 ± 8.2), a moderate-competence group (42.1%, 79.6 ± 6.7), and a high-competence group (10.8%, 95.1 ± 7.9). The model demonstrated high classification accuracy, with an entropy value of 0.948, and the Lo–Mendell–Rubin likelihood ratio test (LMRT) was significant (p = 0.006). Spiritual care competence was significantly positively correlated with spiritual care cognition (r = 0.540) and sense of meaning in life (r = 0.479) (both p < 0.01). Ordinal logistic regression indicated that lack of clinical internship experience, distant teacher–student relationships, absence of humanistic care education, lower levels of spiritual care cognition, and lower sense of meaning in life were key predictors of membership in lower competence profiles.

**Conclusion:**

The spiritual care competence of undergraduate nursing students in China is underdeveloped and urgently needs to be improved. Their potential profiles can be divided into three categories, namely C1-low spiritual care competence, C2-medium spiritual care competence and C3-high spiritual care competence. Clinical internships, effective teacher–student mentoring, humanistic education, as well as individual spiritual cognition and sense of meaning in life, are key facilitators of competence development. It is recommended to integrate structured spiritual care training into undergraduate nursing curricula and to establish a systematic mentorship program.

## Introduction

Spirituality, derived from Latin spiritualitatem and French spiritualité [1], denotes “spiritual quality”. Puchalski et al.[2] define it as a dynamic human essence through which individuals seek ultimate meaning, purpose, and transcendence, fostering connections with self, others, nature, and the sacred. In 1998, the WHO recognized spiritual health as integral to well-being, advocating comprehensive physical, psychological, social, and spiritual care [3]. Modern nursing emphasizes holistic care spanning physical, psychological, social, and spiritual dimensions. Spirituality directly impacts overall health and constitutes a core dimension of nursing practice [4]. Global industrialization, urbanization, aging populations, and disease burden exacerbate patients’ spiritual distress alongside physical suffering [5]. Evidence confirms spiritual care’s efficacy in enhancing well-being among cancer patients [6], chronically ill individuals [5], and the elderly [7]. It mitigates negative emotions (e.g., fear, anxiety) by providing coping mechanisms [8].

Nurses, as frontline caregivers, are pivotal in identifying spiritual crises and delivering such care-a domain distinct from psychological, social, or religious support [9]. Spiritual care competence refers to nurses’ comprehensive ability to assess and address patients’ spiritual needs based on specific knowledge, attitudes, and skills, thereby promoting patients’ psychological resilience and quality of life [10,11]. However, both domestic and international studies indicate that nurses and nursing students generally lack sufficient competence in this domain, primarily due to the absence of systematic courses in pre-service education [12]. In China, the development of spiritual care education faces three major challenges: (1) the lack of a national competency framework. (2) Inadequate clinical mentorship due to the heavy workload of preceptors [13]. (3) The long-standing emphasis on technical skills over humanistic care in medical education. As a result, nursing students often feel ill-equipped to address deep existential suffering when caring for critically ill patients or those facing end-of-life situations, leading to role conflicts [13].

Accordingly, this study adopts an integrative perspective to construct a conceptual model incorporating social learning theory and sociocultural contextual factors. We posit that undergraduate nursing students’ spiritual care competence primarily develops through observational learning and role modeling by mentors in the clinical environment. This process is moderated by individual-level factors, such as spiritual cognition and sense of meaning in life, and is deeply embedded within specific sociocultural contexts, including Confucian ideals of benevolence and Taoist views of nature in traditional Chinese culture. The framework aims to systematically explain the interplay among personal, educational, and environmental factors in the development of competence.

Latent profile analysis is an individual-centered method that uses the probability model to estimate and compare the probability, fit indicators and statistically test to determine each category. Being able to identify different characteristics among individuals through different answers of scale entries, classify samples based on different characteristics, judge the subgroups included in the sample group, and test the relationship between variables in different groups, which is helpful to explore the characteristics and influencing factors of different categories of people [14]. Therefore, this study takes into account the group heterogeneity of spiritual care competence, and discusses the different categories of spiritual care competence of undergraduate nursing students based on the potential profile analysis, and analyzes the influencing factors.

## Research questions

There are 2 research questions:

(1) What is the current situation of the spiritual care competence of undergraduate students in China? How many potential categories exist?
(2) What are the main factors affecting the spiritual care competence of undergraduate nursing students in China?

### Methods

#### Design.

This study is a cross-sectional survey.

#### Participants.

Selection of undergraduate nursing students registered in the Guanzhong region of Shaanxi Province. (1) Inclusion criteria: ① Undergraduate nursing students enrolled and registered in universities in the Guanzhong region of Shaanxi Province; ② Students with clear self-awareness and informed consent voluntarily participate in the survey. (2) Exclusion criteria: ① Students on leave, transferred majors, or about to drop out. ② Students who self-reported diagnosed psychological disorders (e.g., depression, mania, bipolar disorder) in the demographic questionnaire.

#### Variables and Instrument.

(1) Demographic survey questionnaire: After reviewing literature, the research team drafted a demographic survey questionnaire for participants, including 14 items such as age, gender, preference for nursing profession, internship status, and religious beliefs of undergraduate nursing students.
(2) The Chinese Version of the Spiritual Care Competence Scale (C-SCCS): The C-SCCS is a 22-item Chinese adaptation of the Spiritual Care Competence Scale originally developed by van Leeuwen et al.[15] and subsequently translated and revised by Wei et al.[16]. The scale encompasses six dimensions: assessment and implementation, professional development and quality improvement, support, referral, attitudes toward patients’ spirituality, and communication. Each item is rated on a 5-point Likert scale, with total scores ranging from 22 to 110. The overall Cronbach’s α for the Chinese version has been reported as 0.974. In the present study, the scale demonstrated excellent internal consistency, with a total Cronbach’s α of 0.931, indicating high reliability and validity.
(3) Spiritual Care Giving Scale (SCGS): The SCGS was originally developed by Tiew et al.[17] and comprises 35 items across six dimensions, rated on a 6-point Likert scale. The Chinese version (C-SCGS), translated and adapted by Hu et al.[18], includes four dimensions: characteristics of spiritual care, definitions of spirituality and spiritual care, spiritual cognition, and values of spirituality and spiritual care, totaling 34 items. Each item is rated on a 6-point Likert scale, yielding a total score range of 34–204. The scale demonstrates good reliability and validity. In the present study, the C-SCGS showed excellent internal consistency, with a total Cronbach’s α of 0.901, indicating high reliability and validity.
(4) The Meaning in Life Questionnaire (MLQ): The MLQ was developed by Steger et al.[19], and the Chinese version was translated and adapted by Liu Sisi et al.[20]. The scale comprises two subscales: the Presence of Meaning subscale (MLQ-P) and the Search for Meaning subscale (MLQ-S), with a total of nine items. Items are rated on a 7-point Likert scale, yielding total scores ranging from 9 to 63. Scores are categorized as follows: high (>80), indicating clear life goals and meaning. Moderate (66–80), indicating some uncertainty about life meaning and goals. Low (<66), indicating a significant lack of sense of life meaning. The original scale reported a total Cronbach’s α of 0.71. In the present study, the MLQ demonstrated good reliability, with a total Cronbach’s α of 0.803.

### Data collection

#### Phase 1: Establish a research group to conduct a literature review.

(1) Set up a research group consisting of 2 nursing teachers and 5 graduate students. Based on an extensive literature review, the research team will determine the research topic and content based on the current situation of higher nursing education in Shaanxi Province.
(2) Selection of research instrument: According to the literature review, search for research tools according to research variables, and select the appropriate scale for this research according to the research purpose and scope of application of the research tool. Finally, the self-designed questionnaire is combined with the scale used by each variable to form the final questionnaire.

#### Phase 2: Distribute and collect questionnaires.

(1) Pre survey stage: Convenience sampling method was used to select 100 nursing students from a medical college in Shaanxi Province for pre survey. Before the investigation, the investigator first applied to the head of the nursing department of the university for this pre investigation, and sent the formulated investigation questions to the experts for approval before starting this pre investigation. A counselor sent the QR code of the questionnaire to each class’ s WeChat group, inviting students to fill it out. The respondents followed the principle of anonymity and voluntariness to fill out the questionnaire.
(2) According to the research objectives, a purposive sampling method was used to select 1224 undergraduate nursing students from the Guanzhong region of Shaanxi Province, including four undergraduate nursing colleges located in the central area of the region. Before distributing the questionnaire, contact the nursing department heads of four universities and send the survey questionnaire to their respective department heads for review via email, WeChat, QQ, and other means. If there are any issues, provide feedback and suggestions for modification. The research team will make revisions based on the questions raised and send the revised questionnaire to the relevant department heads for review.
(3) After approval, the survey questionnaire was distributed online via the electronic questionnaire platform (Questionnaire Star). Prior to accessing the questionnaire, participants were required to read a detailed digital informed consent form outlining the study purpose, voluntary nature of participation, anonymity and confidentiality measures, data storage policies, and the right to withdraw at any time without penalty. Participants provided explicit consent by selecting an “I agree to participate” checkbox before proceeding. Counselors assisted only in disseminating the survey link via WeChat class groups and were unaware of questionnaire content or individual participation status. The survey questionnaire was collected from July 2024 to February 2025.

### Statistical analysis

The collected data will be subjected to statistical analysis using SPSS27.0 software, and combined with latent profile analysis using Mplus8.0 software.

(1) Latent Profile Analysis (LPA): Latent profile analysis was conducted using the scores of the 22 items of the Spiritual Care Competence Scale as observed variables. Model estimation was performed using maximum likelihood estimation with robust standard errors. To avoid convergence on local maxima, 500 random sets of starting values were specified, followed by 50 final-stage optimizations. Models with one to five latent profiles were fitted sequentially. The optimal number of profiles was determined based on a comprehensive evaluation of the following criteria: the Akaike Information Criterion (AIC), Bayesian Information Criterion (BIC), and sample-size adjusted BIC (with lower values indicating better fit); entropy (with values > 0.80 indicating high classification accuracy); p-values from the likelihood ratio test and the bootstrap likelihood ratio test (p < 0.05 indicating that a k-profile model fits significantly better than a k-1-profile model). In addition, the theoretical relevance and practical interpretability of class proportions were considered in selecting the final model.
(2) Profile Differences and Analysis of Predictive Factors: One-way analysis of variance (ANOVA) or chi-square tests were used to compare differences in demographic characteristics and study-related variables across the latent profiles. Taking latent profile membership (treated as an ordered variable: low, moderate, and high) as the dependent variable, an ordinal multinomial logistic regression model (proportional odds model) was subsequently constructed. The key advantage of this approach lies in its ability to incorporate the ordinal nature of the outcome by estimating a single set of regression coefficients to explain how independent variables influence the cumulative probability of belonging to a higher competence category (e.g., moving from “low” to “moderate or high”). This modeling strategy yields interpretations that are more consistent with the underlying “competence gradient” implied by the latent profiles and provides more precise and robust estimates than multinomial logistic regression, which treats profile membership as a nominal outcome.The BCH method [21] handled “categorical covariates”.

### Ethical considerations

This study was reviewed and approved by the Ethics Committee of School of Medicine, Shaanxi Institute of International Trade & Commerce (Approval No.: SUITC-IRB-2024–0608; Date: 08/06/2024). All procedures adhered to the Declaration of Helsinki.

## Results

### ANOVA results of spiritual care ability of undergraduate nursing students

ANOVA results indicated that age, only-child status, family location, internship experience, relationships with parents, peers, and teachers, receipt of humanistic care education, daily study time, religious beliefs, sense of meaning in life, and spiritual care cognition significantly predicted latent profile membership of spiritual care competence (all p < 0.05). Scores for sense of meaning in life and spiritual care cognition differed significantly across the identified spiritual care competence profiles (Table 1). (See Supplementary Materials).

**Table 1:** ANOVA analysis of three potential profiles of demographic data and spiritual care competence (n = 1224) ) .

items	Number (%)	Potential profile category			*P* vaule
		C1 (%)	C2 (%)	C3 (%)	
Age (years)					
<22	983 (80.3)	456 (79.0)	408 (79.4)	119 (88.5)	<0.011
≥22	241 (19.7)	121 (21.0)	106 (20.6)	14 (10.5)	
Is he/she an only child					
no	383 (31.3)	164 (28.4)	158 (30.7)	61 (45.9)	<0.001
yes	841 (68.7)	413 (71.6)	356 (69.3)	72 (54.1)	
Is the household located in an urban area					
no	341 (27.9)	142 (24.6)	136 (26.5)	63 (47.4)	<0.001
yes	883 (72.1)	435 (75.4)	378 (73.5)	70 (52.6)	
Is it an internship					
no	385 (31.5)	287 (74.5)	92 (23.9)	6 (1.6)	<0.001
yes	839 (68.5)	427 (74.0)	345 (67.1)	127 (50.4)	
Relationship with parents					
intimate	1111 (90.9)	505 (87.5)	482 (93.8)	126 (94.7)	<0.001
Not intimate	111 (9.1)	72 (12.5)	32 (6.2)	7 (5.3)	
Relationship with peers					
intimate	1105 (90.3)	511 (88.6)	467 (90.9)	127 (95.5)	0.029
Not intimate	119 (9.7)	66 (11.4)	47 (9.1)	6 (4.5)	
Relationship with teachers					
intimate	712 (58.2)	285 (49.4)	327 (63.6)	100 (75.2)	<0.001
Not intimate	512 (41.8)	292 (50.6)	187 (36.4)	33 (24.8)	
Have you received humanistic care education					
no	50 (4.1)	32 (5.5)	12 (2.3)	6 (4.5)	0.022
yes	1174 (95.9)	545 (94.5)	502 (97.7)	127 (95.5)	
sDaily study time					
Study time>Rest time	514 (42.0)	226 (39.2)	219 (42.6)	69 (51.9)	0.008
Study time<Rest time	348 (28.4)	181 (31.4)	145 (28.2)	22 (16.5)	
Study time=Rest time	362 (29.6)	170 (29.5)	150 (29.2)	42 (31.6)	
Do you have any religious beliefs					
no	403 (32.9)	177 (30.7)	159 (30.9)	67 (50.4)	<0.001
yes	821 (67.1)	400 (69.3)	355 (69.1)	66 (49.6)	
meaning in life [χ ± s, score]	42.57 ± 8.66	39.48 ± 7.49	43.82 ± 7.46	51.15 ± 10.57	<0.001
Spiritual care cognition [χ±s, score]	162.74 ± 26.76	151.60 ± 26.76	168.35 ± 20.76	189.36 ± 21.36	<0.001

1) C1- low spiritual care competence, C2 -medium spiritual care competence, C3- high spiritual care competence.

### Scores for undergraduate nursing students’ spiritual care competence, spiritual care cognition, and sense of meaning in life

The spiritual care competence of undergraduate nursing students is (77.16 ± 14.61) points, which is at a moderate level. The cognitive sense of spiritual care is (162.74 ± 26.76) points, which is at a moderate level compared. The sense of meaning in life is (42.57 ± 8.66) points, which is at a low level. Please refer to Table 2 for details.

**Table 2:** Scores of spiritual care competence, spiritual care cognition and sense of life significance (n = 1224) ) .

varible	Number of entries	Maximum (scores)	Minimum (scores)	χ ± s (scores)
spiritual care competence	22	22	110	77.61 ± 14.61
spiritual care cognition	34	34	204	162.74 ± 26.76
sense of life significance	9	6	30	42.57 ± 8.66

### Correlation results of spiritual care competence, spiritual care cognition, and sense of meaning in life

The research results showed that a correlation coefficient of r = 0.540, P < 0.01 between spiritual care competence and spiritual care cognition. Spiritual care competence and sense of meaning in life (r = 0.479, P < 0.01). The cognitive sense of spiritual care and the sense of meaning in life (r = 0.487, P < 0.01). Please refer to Table 3 for details. (See Supplementary Materials).

**Table 3:** Correlation between spiritual care competence, spiritual care cognition, and sense of meaning of life(r).

varible	1	2	3	4	5	6	7	8	9
1	–								
2	0.836^*^	–							
3	0.727^*^	0.865^*^	–						
4	0.655^*^	0.764^*^	0.875^*^	–					
5	0.577^*^	0.616^*^	0.610^*^	0.587^*^	–				
6	0.366^*^	0.377^*^	0.393^*^	0.375^*^	0.513^*^	–			
7	0.860^*^	0.930^*^	0.923^*^	0.856^*^	0.790^*^	0.545^*^	–		
8	0.451^*^	0.499^*^	0.472^*^	0.410^*^	0.475^*^	0.346^*^	0.540^*^	–	
9	0.395^*^	0.450^*^	0.446^*^	0.417^*^	0.343^*^	0.340^*^	0.479^*^	0.487^*^	–

Note: 1–9 respectively indicate the evaluation and implementation competence, professional development and quality improvement competence, support and assistance competence, referral service competence, cognitive attitude towards the patient’s spirituality, communication competence, total score of spiritual care competence, total spiritual care cognition, total sense of life meaning score. ^#^ *P* < 0.05, ^*^ *P* < 0.01.

### Potential profile analysis results of spiritual care competence

**Fig 1:**
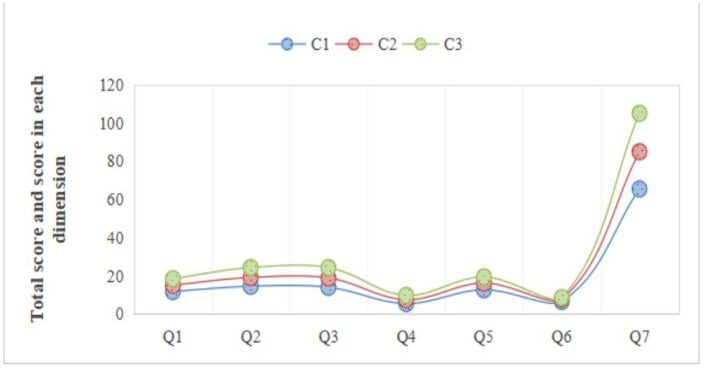
Analysis of the potential profile of the spiritual care competence of undergraduate nursing students. Note: C1- low spiritual care competence, C2 -medium spiritual care competence, C3- high spiritual care competence; Q1-Q7 respectively indicate the evaluation and implementation competence, professional development and quality improvement competence, support and assistance competence, referral service competence, cognitive attitude towards the patient’s spirituality, communication competence, total score of spiritual care competence.

This study started from the basic model and gradually constructed five models with different numbers of profiles. The specific fitting index data is shown in Table 4. The fit indices of the LPA models are presented in Table 2. Based on a comprehensive evaluation of the model fit indices, the AIC, BIC, and aBIC values decreased progressively as the number of profiles increased. When a three-profile solution was specified, the entropy reached a high value of 0.948, and the LMRT was statistically significant (p = 0.006), indicating that the three-profile model provided a significantly better fit than the two-profile model. Although the four- and five-profile solutions showed slight improvements in some fit indices, each of these models produced two classes with proportions below 10%, suggesting limited practical discrimination and insufficient theoretical interpretability. Therefore, the three-profile model was ultimately selected as the optimal solution in this study. C1 (Low): Mean SCCS = 62.3 ± 8.2; C2 (Moderate): 79.6 ± 6.7; C3 (High): 95.1 ± 7.9. Distinctive features: C3 had higher religious affiliation (50.4% vs 30.7% in C1, χ² = 19.5, p < 0.001). Refer to Fig 1.

**Table 4:** Potential profile analysis and fitting index results of spiritual care competence.

Number of sections	AIC	BIC	aBIC	Entropy	LMR	BLRT	Category probability
1	45502.272	45573.810	45529.341	–	–	–	–
2	41392.847	41505.264	41435.383	0.924	<0.001	<0.001	0.5589/0.441
3	39789.683	39942.979	39847.687	0.948	0.006	<0.001	0.471/0.421/0.108
4	37574.026	37768.202	37647.498	0.967	0.017	<0.001	0.050/0.477/0.378/0.096
5	36798.455	37033.510	36887.394	0.952	0.003	<0.001	0.041/0.365/0.305/0.196/0.092

### Multi factor analysis results of spiritual care ability of undergraduate nursing students

Taking spiritual care competence latent profiles (C1 = 1, C2 = 2, C3 = 3) as an ordered dependent variable, variables that were statistically significant in the univariate analysis were entered as independent variables into an ordinal logistic regression model. The results indicated that lack of internship experience, distant relationships with teachers, absence of humanistic care education, lower sense of meaning in life, and lower spiritual care cognition were the main factors associated with membership in lower competence profiles. The test of parallel lines confirmed that the proportional odds assumption was met (p < 0.01). Detailed results are presented in Table 5.

**Table 5:** Orderly and multi-classified logistic regression analysis of the factors affecting spiritual care competence (n = 1224).

varible	*B*	*SE*	*Wald* χ^2^	*OR (*95%*CI)*	*P* vaule
C1	8.788	0.568	239.562		
C2	11.682	0.621	353.444		
No internship (“Yes” as the reference category)	−0.453	0.233	3.793	0.64(0.41 ~ 1.00)	0.049
Close relationship with teachers (“Unclose” as the reference category)	0.323	0.134	5.835	1.38(1.06 ~ 1.79)	0.016
Did not receive humanistic care education (“Received” as the reference category)	−0.759	0.334	5.167	0.47(0.24 ~ 0.90)	0.023
Sense of meaning of life	0.087	0.009	89.238	1.09(1.07 ~ 1.11)	<0.001
Spiritual care cognition	0.031	0.003	111.574	1.03(1.03 ~ 1.04)	<0.001

## Discussion

### Profile characteristics of spiritual care competence among undergraduate nursing students

This study used individual-centered latent profile analysis to examine heterogeneity in spiritual care competence among undergraduate nursing students. Based on model fit indices and interpretability, three profiles were identified: low competence (C1), moderate competence (C2), and high competence (C3). This classification differs from previous international studies and from latent profile analyses among staff in Chinese elderly care institutions [22], suggesting that developmental patterns of spiritual care competence may vary across populations and educational contexts. Unlike Western models that conceptualize spiritual care competence as a linear continuum of knowledge, attitudes, and skills [23], our findings indicate a nonlinear pattern among Chinese nursing students, shaped by interactions between clinical socialization and internalized value systems. In the high-competence group (C3), over half of the students (50.4%) integrated illness experiences with broader life meaning, forming stable meaning-making frameworks that supported spiritual assessment and care in clinical practice [24].

The C1 was primarily characterized by limited clinical exposure, with 74.5% lacking internship experience. This lack of practicum restricted opportunities for observational learning of spiritual care behaviors, consistent with Bandura’ s social learning theory [25,26]. Additionally, more than half of these students (50.6%) reported distant relationships with faculty, reducing instructional scaffolding needed to translate abstract spiritual concepts into practical skills. These factors align with regression results, which identified internship status and faculty relationships as significant predictors of competence level. Students in the C2 generally had clinical exposure (67.1% with internships) but showed incomplete development. Only 63.6% reported close faculty relationships, which may have limited their acquisition of advanced skills, such as assessing existential distress, known to be mentorship-dependent in specialized settings like oncology nursing [27]. The C3 benefited from extensive clinical immersion (94.7% internship completion), strong faculty support, and higher spiritual cognition and sense of life meaning. These students effectively leveraged mentorship and clinical experience to transform observational learning into practiced competence through repeated application of spiritual assessment and care strategies [28]. Regression analyses confirmed that spiritual care cognition and life meaning were independent predictors of profile membership (Table 5), emphasizing their central role. Overall, these findings suggest that undergraduate nursing students’ spiritual care competence depends not only on clinical exposure or education but on the integrated effects of experiential learning, mentorship, and internalized meaning-making.

### Cross-cultural comparative analysis of factors influencing spiritual care competence in cultural contexts

The findings of this study indicate that the lack of clinical internship experience is a major barrier to developing spiritual care competence among undergraduate nursing students. Clinical practicum is a critical stage in nursing education, allowing students to engage with real clinical cases, including patients with severe illness or at the end of life, where spiritual care needs are most apparent [29]. Students without internship experience were less exposed to these contexts and were therefore less able to recognize or respond to patients’ spiritual needs. In the Chinese educational context, internships contribute not only to technical skill acquisition but also to observational learning. Clinical instructors serve as role models, demonstrating empathic communication and compassionate care in daily practice. Consistent with social learning theory, students acquire spiritual care competence by observing and internalizing these behaviors during clinical placements [30]. Students lacking such experiences showed reduced sensitivity to spiritual distress, lower ability to identify existential concerns, and insufficient competence to provide spiritual care, explaining their higher likelihood of being classified in the low-competence profile.

Faculty-student relationships also significantly influenced spiritual care competence. Students reporting closer relationships with instructors were more likely to demonstrate moderate to high levels of competence. Close pedagogical interactions allow instructors to guide students through real or simulated care scenarios, supporting the translation of abstract concepts into clinical practice [31,32]. These results highlight the role of effective mentorship in strengthening students’ understanding and application of spiritual care. Humanistic education was also identified as an essential component. As a core aspect of holistic nursing, spiritual care requires educational approaches beyond technical training [33,34]. Previous research shows that students who participate in humanities or philosophy courses demonstrate higher spiritual health and reflective capacity [22], which may enhance their ability to interpret patient suffering and provide appropriate spiritual support. Overall, these findings suggest that spiritual care competence among undergraduate nursing students is shaped by the combined effects of clinical exposure, faculty mentorship, and humanistic education. Nursing programs should integrate spiritual care training into clinical practica and humanistic curricula, using case-based learning and guided reflection to help students develop spiritual care as a core component of holistic nursing practice.

### Culturally embedded linkages between spiritual cognition and meaning in life

The results of this study show significant positive correlations among spiritual care cognition, meaning in life, and spiritual care competence, consistent with previous research [35,36]. Multivariate regression further identified spiritual care cognition and meaning in life as key determinants of nursing undergraduates’ spiritual care competences. First, nurses’ spiritual cognition directly influences their ability to assess and manage patients’ spiritual needs. Enhanced cognition allows for accurate identification of existential concerns and facilitates the delivery of high-quality spiritual interventions, which are essential for competent care [37]. In the Chinese context, students’ spiritual cognition emphasizes introspective self-awareness, reflecting Confucian self-cultivation and Taoist reflective practices, rather than connection with a divine entity as commonly emphasized in Western measures [22]. Students with heightened spiritual cognition demonstrate culturally attuned empathy, accurately interpreting patients’ verbal and nonverbal cues, perceiving inner experiences across bio-psycho-spiritual dimensions, and refining their skills through mentorship. This integration of cognitive and emotional processing supports effective spiritual care that combines technical proficiency with relational ethics [36,37].

Second, spirituality is closely linked with the perception of life meaning. As a component of humanistic care competence, students’ spiritual care abilities correlate strongly with their sense of life purpose [33]. C3 students interpret patients’ spiritual challenges within a relational and ethical framework, emphasizing responsibilities to family and community. For example, they may reframe a cancer patient’ s concern about children’s education as part of intergenerational duty. Taoist perspectives, such as viewing adversity as a natural and transformable process, also help students reconstruct patient suffering as meaningful experience, enhancing their own sense of purpose and preventing emotional exhaustion common in Western “savior” models [38]. Overall, students with higher spiritual care cognition and life meaning are better able to recognize spiritual needs, provide targeted interventions, and improve patient well-being while experiencing personal fulfillment. Their awareness of patients’ inner experiences fosters respect for life and reinforces the integration of culturally grounded ethical principles into clinical practice, supporting more effective and contextually sensitive spiritual care.

### Limitations & implications

This study has several limitations. First, the sample was drawn exclusively from nursing students in Shaanxi Province, which may limit the generalizability of the findings to other regions. Second, as a cross-sectional study relying on self-reported data, the results are subject to response biases. Future studies should adopt longitudinal designs to examine causal relationships and strengthen the reliability of conclusions. Ethical approval was obtained, and all procedures followed standard observational study guidelines. The findings underscore the importance of integrating humanistic and scientific elements in spiritual care education. Unlike Western models that often emphasize religious frameworks, China’s approach incorporates Confucian benevolence, Taoist natural harmony, and Buddhist compassion. Internalizing these cultural values, together with clinical socialization, appears to support the development of spiritual care competence.

For curriculum implementation, an observation-reflection-practice-internalization model is recommended. Students can engage in case analyses demonstrating Confucian benevolent care, participate in Taoist-inspired workshops on acceptance of life and death, and learn through guided modeling by trained clinical instructors. Complementary practices, such as Confucian self-reflection and Taoist contemplative exercises, may further enhance empathy and ethical sensitivity. This integrated approach facilitates the development of spiritual care competence throughout patient engagement from initial contact and relationship-building to intervention delivery that establishing a culturally grounded, non-religious framework that may inform global humanistic nursing education.

## Conclusion

Spiritual care competence among Chinese nursing undergraduates remains suboptimal and can be classified into three profiles: low, moderate, and high. Its development is strongly influenced by clinical internships, faculty-student relationships, humanistic education, spiritual cognition, and sense of life meaning. To address these factors systematically, integrating mandatory spiritual care training into the undergraduate curriculum is essential, alongside a structured faculty-student mentorship system. Standardized courses can provide foundational knowledge, embedding Confucian concepts of benevolence and Taoist principles of natural harmony into spiritual assessment protocols. Simultaneously, the mentorship program allows clinical instructors to model context-specific spiritual care behaviors during internships, helping students bridge the gap between theory and practice. Through guided reflective practice, students internalize cultural values while enhancing skills in identifying existential distress and supporting patients’ meaning-making processes. Implementing these dual strategies can promote the development of holistic competence, transforming spiritual care from a set of fragmented skills into an integrated clinical practice philosophy aligned with Chinese humanistic healthcare principles.

## Supporting information

S1 Table Detailed demographic characteristics of the study participants and results of univariate analyses (n = 1,224). (DOCX)

S2 Table Correlation matrix (r values) among the dimensions of spiritual care competence, cognition, and sense of meaning in life. (DOCX)
