# Supplementary material for: Latent profiles of spiritual care competence among Chinese nursing undergraduates: Correlations with spiritual care cognition and meaning of life
Source: PLoS One. 2026 Feb 6;21(2):e0342051. doi: 10.1371/journal.pone.0342051 (PMC12880687; doi:10.1371/journal.pone.0342051)
Supplement: S1 Table — (DOCX) [file pone.0342051.s001.docx]

Table S1 Detailed demographic characteristics of the study participants and results of univariate analyses（n=1224）

| items |  | Number （%） | Potential profile category | |  | χ^2^ /F | *P* vaule |
| --- | --- | --- | --- | --- | --- | --- | --- |
|  |  |  | C1（%） | C2（%） | C3（%） |  |  |
| Age (years) | ＜22 | 983（80.3） | 456（79.0） | 408（79.4） | 119（88.5） | 9.050^1）^ | ＜0.011 |
|  | ≥22 | 241（19.7） | 121（21.0） | 106（20.6） | 14（10.5） |  |  |
|  |  |  |  |  |  |  |  |
| Is he/she an only child | no | 383（31.3） | 164（28.4） | 158（30.7） | 61（45.9） | 14.640^1）^ | ＜0.001 |
|  | yes | 841（68.7） | 413（71.6） | 356（69.3） | 72（54.1） |  |  |
|  |  |  |  |  |  |  |  |
| Is the household located in an urban area | no | 341（27.9） | 142（24.6） | 136（26.5） | 63（47.4） | 26.353^1）^ | ＜0.001 |
|  | yes | 883（72.1） | 435（75.4） | 378（73.5） | 70（52.6） |  |  |
|  |  |  |  |  |  |  |  |
| Is it an internship | no | 385（31.5） | 287（74.5） | 92（23.9） | 6（1.6） | 27.648^1）^ | ＜0.001 |
|  | yes | 839（68.5） | 427（74.0） | 345（67.1） | 127（50.4） |  |  |
|  |  |  |  |  |  |  |  |
| Relationship with parents | intimate | 1111（90.9） | 505（87.5） | 482（93.8） | 126（94.7） | 15.679^1）^ | ＜0.001 |
|  | Not intimate | 111（9.1） | 72（12.5） | 32（6.2） | 7（5.3） |  |  |
|  |  |  |  |  |  |  |  |
| Relationship with peers | intimate | 1105（90.3） | 511（88.6） | 467（90.9） | 127（95.5） | 7.082^1）^ | 0.029 |
|  | Not intimate | 119（9.7） | 66（11.4） | 47（9.1） | 6（4.5） |  |  |
|  |  |  |  |  |  |  |  |
| Relationship with teachers | intimate | 712（58.2） | 285（49.4） | 327（63.6） | 100（75.2） | 41.224^1）^ | ＜0.001 |
|  | Not intimate | 512（41.8） | 292（50.6） | 187（36.4） | 33（24.8） |  |  |
|  |  |  |  |  |  |  |  |
| Have you received humanistic care education | no | 50（4.1） | 32（5.5） | 12（2.3） | 6（4.5） | 7.627^1）^ | 0.022 |
|  | yes | 1174（95.9） | 545（94.5） | 502（97.7） | 127（95.5） |  |  |
|  |  |  |  |  |  |  |  |
|  |  |  |  |  |  |  |  |
| Daily study time | Study time>Rest time | 514（42.0） | 226（39.2） | 219（42.6） | 69（51.9） | 13.751^1）^ | 0.008 |
|  | Study time＜Rest time | 348（28.4） | 181（31.4） | 145（28.2） | 22（16.5） |  |  |
|  | Study time＝Rest time | 362（29.6） | 170（29.5） | 150（29.2） | 42（31.6） |  |  |
|  |  |  |  |  |  |  |  |
| Do you have any religious beliefs | no | 403（32.9） | 177（30.7） | 159（30.9） | 67（50.4） | 19.482^1）^ | ＜0.001 |
|  | yes | 821（67.1） | 400（69.3） | 355（69.1） | 66（49.6） |  |  |
|  |  |  |  |  |  |  |  |
| meaning in life  [χ±s，score] |  | 42.57±8.66 | 39.48±7.49 | 43.82±7.46 | 51.15±10.57 | 129.961^2）^ | ＜0.001 |
|  |  |  |  |  |  |  |  |
| Spiritual care cognition  [χ±s，score] |  | 162.74±26.76 | 151.60±26.76 | 168.35±20.76 | 189.36±21.36 | 160.197^3）^ | ＜0.001 |

1. χ^2^ 值；2）F值。C1- low spiritual care competence, C2 -medium spiritual care competence,

C3- high spiritual care competence
