# Supplementary material for: Latent profiles of spiritual care competence among Chinese nursing undergraduates: Correlations with spiritual care cognition and meaning of life
Source: PLoS One. 2026 Feb 6;21(2):e0342051. doi: 10.1371/journal.pone.0342051 (PMC12880687; doi:10.1371/journal.pone.0342051)
Supplement: S2 Table — (DOCX) [file pone.0342051.s002.docx]

Table S2 Correlation matrix (r values) among the dimensions of spiritual care competence, cognition, and sense of meaning in life

| varible | 1 | 2 | 3 | 4 | 5 | 6 | 7 | 8 | 9 | 10 | 11 | 12 | 13 | 14 | 15 |
| --- | --- | --- | --- | --- | --- | --- | --- | --- | --- | --- | --- | --- | --- | --- | --- |
| 1 | - |  |  |  |  |  |  |  |  |  |  |  |  |  |  |
| 2 | 0.836* | - |  |  |  |  |  |  |  |  |  |  |  |  |  |
| 3 | 0.727* | 0.865* | - |  |  |  |  |  |  |  |  |  |  |  |  |
| 4 | 0.655* | 0.764* | 0.875* | - |  |  |  |  |  |  |  |  |  |  |  |
| 5 | 0.577* | 0.616* | 0.610* | 0.587* | - |  |  |  |  |  |  |  |  |  |  |
| 6 | 0.366* | 0.377* | 0.393* | 0.375* | 0.513* | - |  |  |  |  |  |  |  |  |  |
| 7 | 0.860* | 0.930* | 0.923* | 0.856* | 0.790* | 0.545* | - |  |  |  |  |  |  |  |  |
| 8 | 0.447* | 0.485* | 0.451* | 0.382* | 0.472* | 0.346* | 0.526* | - |  |  |  |  |  |  |  |
| 9 | 0.433* | 0.493* | 0.482* | 0.440* | 0.433* | 0.382* | 0.530* | 0.889* | - |  |  |  |  |  |  |
| 10 | 0.403* | 0.441* | 0.414* | 0.342* | 0.448* | 0.330* | 0.484* | 0.865* | 0.878* | - |  |  |  |  |  |
| 11 | 0.421* | 0.417* | 0.444* | 0.390* | 0.446* | 0.309* | 0.506* | 0.854* | 0.886* | 0.919* | - |  |  |  |  |
| 12 | 0.451* | 0.499* | 0.472* | 0.410* | 0.475* | 0.346* | 0.540* | 0.963* | 0.956* | 0.944* | 0.948* | - |  |  |  |
| 13 | 0.387* | 0.437* | 0.427* | 0.395* | 0.341* | 0.339* | 0.466* | 0.460* | 0.458* | 0.411* | 0.418* | 0.463* | - |  |  |
| 14 | 0.351* | 0.404* | 0.408* | 0.386* | 0.299* | 0.295* | 0.430* | 0.443* | 0.443* | 0.392* | 0.407* | 0.447* | 0.743* | - |  |
| 15 | 0.395* | 0.450* | 0.446* | 0.417* | 0.343* | 0.340* | 0.479* | 0.483* | 0.482* | 0.429* | 0.441* | 0.487* | 0.944* | 0.928* | - |

Note: 1-15 respectively indicate the evaluation and implementation competence, professional development and quality improvement competence, support and assistance competence, referral service competence, cognitive attitude towards the patient's spirituality, communication competence, total score of spiritual care competence, spiritual care characteristics, spiritual and spiritual care definition, spiritual cognition, spiritual and spiritual care value, total spiritual care cognition score, sense of life meaning, seeking meaning, total sense of life meaning. ^#^ *P*<0.05, ^*^ *P*<0.01.
